# Supplementary material for: Feasibility of CPAP application and variables related to worsening of respiratory failure in pregnant women with SARS-CoV-2 pneumonia: Experience of a tertiary care centre
Source: PLoS One. 2021 Oct 19;16(10):e0258754. doi: 10.1371/journal.pone.0258754 (PMC8525751; doi:10.1371/journal.pone.0258754)
Supplement: Attachment — Submitted filename: review plos one CPAPA y embarazadas COVID19.docx [file pone.0258754.s002.docx]

Review : Feasibility of CPAP application and factors associated with worsening of respiratory

failure in pregnant women with SARS-CoV-2 pneumonia: experience of a tertiary care

centre

Had been an honor to review an original article titled Feasibility of CPAP application and factors associated with worsening of respiratory failure in pregnant women with SARS-CoV-2 pneumonia: experience of a tertiary care centre”, for the initially congratulate to authors by their effort during the COVID-19 pandemic

About the article, I have an observation: the authors used the term ““ASSOCIATED FACTORS” however, the análisis plan performed don’t show any statistical association but means the difference between groups

I recommend performing a logistic regression (univariate and bivariate) or another analysis to justify the association. In my criteria, this would be valuable information to accept this article
