# Supplementary material for: Feasibility of CPAP application and variables related to worsening of respiratory failure in pregnant women with SARS-CoV-2 pneumonia: Experience of a tertiary care centre
Source: PLoS One. 2021 Oct 19;16(10):e0258754. doi: 10.1371/journal.pone.0258754 (PMC8525751; doi:10.1371/journal.pone.0258754)
Supplement: Attachment — Submitted filename: Response to reviewers.doc [file pone.0258754.s003.doc]

Dear Dr Tai-Heng Chen, Academic Editor

*PLOS ONE*

As suggested by the editorial board of PLOS ONE, we are pleased to submit our revised manuscript entitled “Feasibility of CPAP application and factors associated with worsening of respiratory failure in pregnant women with SARS-CoV-2 pneumonia: experience of a tertiary care centre”.

We have addressed all the points very reasonably raised by the Reviewers and amended our manuscript accordingly. We believe this has increased considerably the quality of our article.

Please find below a point-by-point reply to the Reviewers comments, as requested.

We hope our revised manuscript will be considered for publication in the PLOS ONE in its present form.

Sincerely yours

Paola Faverio and Sara Ornaghi (for the Authors)

**Journal Requirements**

1. Please ensure that your manuscript meets PLOS ONE's style requirements, including those for file naming.

We changed the files’ name as per Journal style.

2. We note that you have indicated that data from this study are available upon request. PLOS only allows data to be available upon request if there are legal or ethical restrictions on sharing data publicly.

**Prompts**

a. If there are ethical or legal restrictions on sharing a de-identified data set, please explain them in detail (e.g., data contain potentially sensitive information, data are owned by a third-party organization, etc.) and who has imposed them (e.g., an ethics committee). Please also provide contact information for a data access committee, ethics committee, or other institutional body to which data requests may be sent.
b. If there are no restrictions, please upload the minimal anonymized data set necessary to replicate your study findings as either Supporting Information files or to a stable, public repository and provide us with the relevant URLs, DOIs, or accession numbers.

We uploaded the minimal anonymized data set as requested.

3. Please include your full ethics statement in the ‘Methods’ section of your manuscript file. In your statement, please include the full name of the IRB or ethics committee who approved or waived your study, as well as whether or not you obtained informed written or verbal consent. If consent was waived for your study, please include this information in your statement as well.

The study was approved by the Institutional Review Board of the University of Milano Bicocca (3140/2020). Written consent was obtained by patients included in the study. We added this information in the text.

**Review Comments to the Author**

**Reviewer #1**:

Had been an honor to review an original article titled Feasibility of CPAP application and factors associated with worsening of respiratory failure in pregnant women with SARS-CoV-2 pneumonia: experience of a tertiary care centre”, for the initially congratulate to authors by their effort during the COVID-19 pandemic

We thank the Reviewer for his/her appreciation of our work, highlighting our research effort during the SARS-CoV-2 pandemic.

**R1.C1**

About the article, I have an observation: the authors used the term ““ASSOCIATED FACTORS” however, the análisis plan performed don’t show any statistical association but means the difference between groups

I recommend performing a logistic regression (univariate and bivariate) or another analysis to justify the association. In my criteria, this would be valuable information to accept this article

**R1.R1**

The Referee pointed out that the term ‘associated factors’ has been inaccurately used in the manuscript since the analysis we performed, i.e., univariate, cannot demonstrate an association (between the need of CPAP and patients’ characteristics) but only the presence of differences between the study groups (“room air” or “oxygen only” *versus* CPAP). Thus, the Referee suggested to perform a logistic regression analysis.

We agree with the referee that the term ‘association’ has been used incorrectly. Since this is a retrospective cohort study, statistical analyses can only provide information regarding the presence or not of significant differences between the assessed groups. We have now addressed this point by substituting the term ‘association’ with more statistically appropriate terms throughout the manuscript.

As per performing additional analyses, a logistic regression analysis could be helpful if significant differences observed at the univariate analysis are considered to be biased by differences in patients’ characteristics. In our cohort, patients requiring CPAP had similar demographic and obstetric variables compared to patients in room air or requiring oxygen only, thus preventing an effect of confounding factors on the observed differences in CRP levels and rate of patients with ≥3 lobes involvement. In addition, the small size of the CPAP group (n=10) would substantially limit the validity of a logistic regression analysis.

**Reviewer #2**:

This paper is very interesting and well written. It brings up clinical evolution among COVID-19 pregnant women, especially under the use of helmet CPAP. The study comprised 41 patients, which is a reasonable number in this context. Although retrospective, there is a systematic protocol to be followed in this center, which makes it possible to draw important conclusions.

The use of chest x-ray instead of computed tomography probably underestimated the extension of the disease, but I think this is acceptable due to the pregnancy.

Some points of concern:

**R2.C1**

Why should one expect that helmet CPAP wouldn’t be feasible to apply in a pregnant woman? Make a comment regarding this issue in the introduction.

**R2.R1**

We thank the Reviewer for rising this point. In the available literature, evidence supports the feasibility of helmet CPAP application in adult patients with community acquired pneumonia (*Cosentini et al. Chest. 2010;138:114-20*). In contrast, no evidence is available regarding helmet CPAP utilization and feasibility in pregnant women with SARS-CoV-2 pneumonia. However, as indicated by the Reviewer, we explained this issue in the introduction of the manuscript.

**R2.C2**

The classic cutoffs to define acute respiratory failure are PaO2 of 60 mmHg and PaCO2 of 50 mmHg. Looking at Table 2, PaO2 interquartile range varied from 77.25 to 85.75 on admission. Patients got worse and probably showed lower values that did not appear at this table because they were on oxygen therapy. In the discussion section, the authors also mentioned moderate to severe ARF. What were the specific criteria used to define and categorize acute respiratory failure?

**R2.R2**

We thank the Reviewer for this comment. None of the patients required oxygen supplementation on hospital admission. While the mean values of PaO2 pre-CPAP (T1) and during CPAP (T2) indicated in Table 2 are obtained in patients receiving oxygen therapy, therefore, in order to evaluate respiratory failure severity, we utilized the PaO2 / FIO2 ratio. This solid instrument allowed us to define respiratory failure severity.

**R2.C3**

How was FIO2 calculated in patients under oxygen therapy by nasal catheter? There are some practical rules, but they could be inaccurate. I think the authors should describe how they did that.

**R2.R3**

All ABG analyses were performed in patients wearing a Venturi mask to obtain a precise FIO2 evaluation. However, during the hospital stay, Venturi mask was alternated to nasal catheter in patients with mild respiratory impairment to improve patients’ comfort.

**R2.C4**

FIO2 (capital letter I) should be written instead of FiO2, according to standardization of definitions and symbols in respiratory physiology (Fed Proc 1950; 9:602-5).

**R2.R4**

We thank the Reviewer for this observation. The term “FiO2” has now been substituted with the term “FIO2” throughout the manuscript.

**R2.C5**

I think that “helmet CPAP” (I suggest h-CPAP or H-CPAP) is advisable whenever you need to refer to it, because “CPAP” is traditionally associated to a face mask.

**R2.R5**

We agree with the Reviewer’s comment and the abbreviation ‘h-CPAP’, as suggested, has now been used.
